# Quantitative Assessment of Portal Hypertension by Two-Dimensional Shear Wave Elastography in Rat Models of Nonalcoholic Fatty Liver Disease: Comparison With Four Composite Scores

**DOI:** 10.3389/fmed.2022.844558

**Published:** 2022-03-31

**Authors:** Bingtian Dong, Yuping Chen, Guorong Lyu, Yongjian Chen, Ran Qin

**Affiliations:** ^1^Department of Ultrasound, The Second Affiliated Hospital of Fujian Medical University, Quanzhou, China; ^2^Department of Endocrinology, The Second Affiliated Hospital of Fujian Medical University, Quanzhou, China; ^3^Department of Clinical Medicine, Quanzhou Medical College, Quanzhou, China; ^4^Department of Ultrasound, Chenggong Hospital, Xiamen University, Xiamen, China

**Keywords:** nonalcoholic fatty liver disease, portal hypertension, diagnosis, noninvasive method, two-dimensional shear wave elastography

## Abstract

**Background:**

Measurement of hepatic venous pressure gradients is the gold standard for assessing portal hypertension (PH) but is invasive with potential complications. We aimed to assess the performance in liver and spleen stiffness measurement (LSM and SSM, respectively) by two-dimensional shear wave elastography (2D-SWE) and composite scores including liver stiffness-spleen diameter to platelet ratio score (LSPS), platelet (PLT) count/spleen diameter ratio (PSR), aspartate aminotransferase (AST)/alanine aminotransferase ratio (AAR), and AST-to-PLT ratio index (APRI) for diagnosing PH in nonalcoholic fatty liver disease (NAFLD) rat models.

**Methods:**

Animal models with PH in NAFLD were established in 65 rats, which then underwent 2D-SWE measurements. Morphological and biological parameters were collected for calculation of four composite scores. Correlations of noninvasive methods with portal venous pressure were evaluated by Spearman correlation analysis. The area under the receiver operating characteristic curve (AUC) was used to assess the performance of noninvasive methods in predicting PH.

**Results:**

LSM and SSM were significantly associated with portal venous pressure (*r* = 0.636 and 0.602, respectively; all *P* < 0.001). The AUCs of LSM and SSM in the diagnosis of PH were 0.906 (95% confidence interval [CI]:0.841–0.97) and 0.87 (95% CI:0.776–0.964), respectively, and were significantly higher than those in composite scores. The AUCs for LSPS, PSR, AAR, and APRI were 0.793, 0.52, 0.668, and 0.533, respectively, for diagnosing PH. The AUCs of the combined models of LSM and SSM, LSM and PLT, SSM and PLT, and LSM, SSM and PLT were 0.923, 0.913, 0.872, and 0.923, respectively. The four combined models showed no statistical differences compared to LSM and SSM in evaluating PH (all *P* > 0.05).

**Conclusions:**

LSM and SSM by 2D-SWE can be used as promising noninvasive parameters for diagnosing PH in NAFLD and have higher accuracy than composite scores. The combined models, compared to LSM and SSM, did not significantly improve the performance in diagnosing PH.

## Introduction

Nonalcoholic fatty liver disease (NAFLD) has become the most common chronic liver disease, with an estimated prevalence of 25% worldwide (1, 2). Notably, approximately one-quarter of NAFLD cases progress into cirrhosis in 10 years and are at an increased risk of developing portal hypertension (PH) (3). PH is defined as pathologically elevated pressure in the portal venous system (4). It can lead to several serious complications associated with advanced NAFLD, including bleeding from gastroesophageal varices, and has high morbidity and mortality (5, 6). Therefore, accurate and timely assessment of PH is crucial to improve prognosis and clinical decision-making.

Traditionally, measurement of hepatic venous pressure gradient (HVPG) remains the gold standard for diagnosing PH (7). It is, however, an invasive and costly procedure that requires a specialized angiographic interventional center as well as skillful measurement by an experienced operator, which greatly hampers its routine use in clinical practice (8). Given the drawbacks of HVPG measurement, considerable effort has been devoted to develop a noninvasive tool that can evaluate and monitor PH (9, 10).

In this regard, development of a noninvasive assessment method by elastography may offer a valuable alternative. Elastography is an imaging method that objectively evaluates tissue stiffness, and has recently been developed for assessment of liver fibrosis stage and PH (11, 12). Two-dimensional shear wave elastography (2D-SWE), a promising novel ultrasound-based elastography technique for quantitatively real-time imaging of tissue stiffness (13), has lower cost, is readily available, and simple to utilize compared with invasive methods (e.g., liver biopsy and HVPG measurement). 2D-SWE combines elastogram with conventional B-mode ultrasonography, so operators can directly visualize the liver for high-quality measurements while performing elastography (14). Importantly, 2D-SWE has high applicability in clinics, and measurements can be performed on patients with ascites (8). As such, this technique is suitable for advanced liver diseases, where PH is the main driver of prognosis (15). In particular, liver stiffness measurement (LSM) obtained by 2D-SWE has recently been demonstrated to predict the degree of fibrosis with good diagnostic performance (16–18). Our previous meta-analysis has shown that 2D-SWE has better diagnostic performance than serum fibrosis biomarkers in predicting liver fibrosis induced by chronic hepatitis B (CHB) (19). In recent years, composite scores combining LSM with other parameters, such as liver stiffness-spleen diameter to platelet ratio score (LSPS), have been developed to evaluate esophageal varices and PH (20). Platelet count (PLT)/spleen diameter ratio (PSR) has also been introduced for diagnosing esophageal varices (21).

Previously, several studies have reported that spleen stiffness measurement (SSM) by transient elastography (TE) can be used for noninvasive assessment of PH (22, 23). However, this technique has some technical limitations; for example, it cannot be used on patients with ascites, which limit its clinical application in advanced liver diseases (8). For patients with obesity or a narrow intercostal space, the applicability of TE may also be limited (15). At present, there are insufficient studies on the diagnostic efficiency of SSM obtained by 2D-SWE in the prediction of PH. Moreover, to the best of our knowledge, the value of 2D-SWE in the evaluation of PH in NAFLD has not yet been investigated and compared to the performance of composite scores.

Thus, in this study, we investigated the diagnostic performance of LSM and SSM obtained by 2D-SWE in predicting PH in NAFLD rat models and compared it with that of the four composite scores. In addition, we also studied four combined models, namely, the LSM and SSM combined model, the LSM and PLT combined model, the SSM and PLT combined model, and the LSM, SSM and PLT combined model, for the diagnosis of PH.

## Materials and Methods

### Animal Model

All the experiments were reviewed and approved by the Institutional Animal Care and Use Committee (IACUC) of The Second Affiliated Hospital of Fujian Medical University.

Male Sprague-Dawley rats (200–250 g) were purchased from Shanghai Laboratory Animal Center (SLAC, Shanghai, China) and housed in sterile isolated cages with a 12:12 light-dark cycle at room temperature (20–25 °C) and relative humidity of 40–60%. Eighty rats were divided into 2 groups. In total, 65 rats were randomly included in the first group and used for the experimental model of NAFLD, and provided a methionine- and choline-deficient (MCD) diet for 12 weeks (24). The MCD diet was obtained from the branch of Dyets Inc. in China (#519580; Wuxi, China). The second group (control group) consisted of 15 rats, which were provided a standard diet with sterilized food and water. NAFLD severity was histologically confirmed.

### Liver and Spleen Stiffness Measured by 2D-SWE

**Figure 1:**
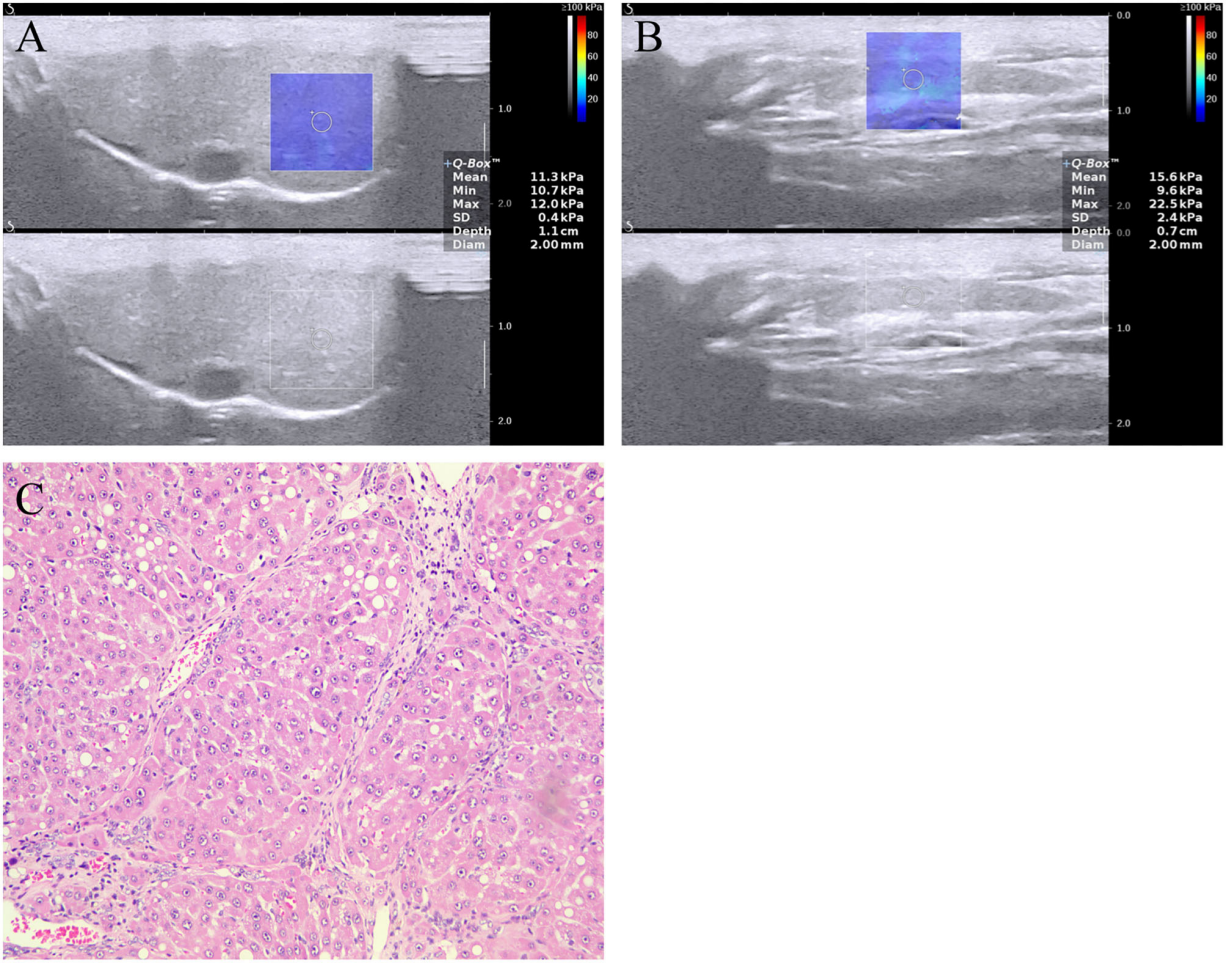
Two-dimensional shear wave elastography (2D-SWE) measurement of the **(A)** liver and **(B)** spleen, and **(C)** image of liver section stained with hematoxylin and eosin in a rat model with NAFLD. The mean LSM and SSM were 11.3 ± 0.4 and 15.6 ± 0.7 kPa, respectively. The image of liver section revealing fibrosis stage F4 (cirrhosis). LSM, liver stiffness measurement; NAFLD, nonalcoholic fatty liver disease; SSM, spleen stiffness measurement.

The rats were fasted overnight before 2D-SWE measurements. LSM and SSM by 2D-SWE were performed using an Aixplorer (Supersonic Imagine, Aix-en-Provence, France) ultrasound imaging system with an SL15-4 transducer. For the rat study, this ultrasound imaging system was set to the superficial (thyroid) imaging mode. 2D-SWE analysis was conducted by an experienced radiologist who was blinded to the results of other diagnostic tests. For 2D-SWE measurements, (1) the sampling frame was set to a 1.5 cm × 1.5 cm which was placed ~1 cm under the liver capsule, avoiding large bile and vessels; and (2) the region of interest had a diameter of 2 mm for LSM and SSM, which was placed in the position of the homogeneous elastographic image signal for quantitative analysis (Figure 1). For each rat, LSM and SSM were considered reliable if the inter-quartile range (IQR)/median value was <30% (25). Five LSMs and SSMs per rat were performed on a defined site, and the median value of five readings was recorded as LSM/SSM expressed in kilopascal (kPa).

### Morphological and Biological Parameters

At the time of ultrasound examination, spleen diameter was determined. Spleen diameter was defined as the maximum spleen bipolar diameter at the level of the splenic hilum (20). Biological parameters, such as PLT, red cell distribution width, aspartate aminotransferase (AST), alanine aminotransferase (ALT), and gamma-glutamyl transferase (GGT), were also collected on the day of ultrasound examination. Four composite scores were calculated as follows: (1) LSPS = LSM (kPa) × spleen diameter (in cm)/PLT (×10^9^/L); (2) PSR = PLT (×10^9^/L)/spleen diameter (in cm); (3)AST/ALT ratio (AAR) = AST (IU/L)/ALT (IU/L); and (4) AST-to-PLT ratio index (APRI) = (AST/upper limit of normal for AST) ×100/PLT (×10^9^/L). Of note, LSPS was calculated in our study according to the same formula described by Kim et al. (20) but by 2D-SWE instead.

### Portal Venous Pressure Measurement

After the rats were fasted overnight, portal venous pressure measurement was performed immediately after 2D-SWE scan. Portal venous pressure was measured using a digital blood pressure analyzer (BL-420F; Techman Software, Chengdu, China) with computer interface. A pressure transducer module (PT-120; Techman Software, Chengdu, China) was connected to the digital blood pressure analyzer (channel 1). Before the portal venous pressure measurement, an anticoagulant citrate dextrose (ACD) solution was used to perfuse this entire setup. The ACD solution was purchased from Macklin (#A885470; Shanghai, China). Calibration of the analyzer was carried out before each reading according to the manufacturer's instructions. Then, a 12-gauge needle was used and inserted into the exposed portal vein of the rats. Finally, we continuously monitored real-time portal venous pressure and recorded it as an average reading.

### Tissue Analyses

After the portal venous pressure measurement, the animals were sacrificed immediately, and liver tissues were harvested, fixed in 10% buffered formalin, and then sliced to a thickness of 5 um for staining with hematoxylin and eosin and Masson's trichrome. In this study, the sections were assessed for severity of lipid infiltration, lobular inflammation, ballooning degeneration, and fibrosis using the semiquantitative scoring system of steatosis, activity, and fibrosis (SAF) (26).

### Statistical Analysis

Statistical analyses were conducted using SPSS version 26.0 (IBM, Armonk, NY, United States) and GraphPad Prism (GraphPad Prism 7.0, United States). For comparison between groups *t*-test or Mann-Whitney U test was performed when appropriate. The intra-operator reliability of 2D-SWE was assessed with intra-class correlation coefficient (ICC), with a value of >0.75 indicating excellent reliability. Moreover, the coefficient of variation (CV) was also calculated. A CV value of 10% or less was considered to indicate good reproducibility. Absolute ICC was used to test the concordance among LSM values calculated as a median of three or five measurements. Similarly, the absolute ICC was calculated to test the concordance among SSM values. Spearman correlation test was conducted in this study to evaluate the correlation between noninvasive methods and portal venous pressure. PH-positive was defined as portal venous pressure ≥5 mmHg, while PH-negative was defined as portal venous pressure <5 mmHg (4). The diagnostic performance of different noninvasive methods in predicting PH was assessed by receiver operating characteristic (ROC) curves. In addition, the four combined models, namely, the LSM and SSM combined model (combined model 1), the LSM and PLT combined model (combined model 2), the SSM and PLT combined model (combined model 3), and the LSM, SSM, and PLT combined model (combined model 4), were also explored by multivariate logistic analysis. Cutoff values were defined using the Youden index. Sensitivity, specificity, positive predictive value (PPV), and negative predictive value (NPV) were calculated. Comparisons of the area under the ROC curves (AUC) were performed using the DeLong test. *P* < 0.05 was considered statistically significant.

## Results

### Baseline Characteristics

Of all the 80 rats, 1 (1.3%) with NAFLD was excluded because of death after anesthesia before laparotomy. Then, the initial study samples included 79 rats. There are 15 controls and a total of 64 rats had NAFLD. The severity of NAFLD was histologically confirmed. The portal venous pressure measurement was performed after modeling, and all the rats with NAFLD had a portal venous pressure value >5 mmHg, indicating successful modeling. For that control rats that were provided a standard diet with sterilized food and water, all portal venous pressure values were <5 mmHg.

Table 1 shows in detail the biological, morphological, and elastography characteristic parameters observed in the rats. When compared with controls, rats with NAFLD had significantly elevated red cell distribution width (*P* = 0.041), ALT (*P* = 0.009), spleen diameter (*P* < 0.001), portal venous pressure (*P* < 0.001), LSM (*P* < 0.001), and SSM (*P* < 0.001). Between the two groups, there were no significant differences in PLT (*P* = 0.419), AST (*P* = 0.333), and GGT (*P* = 0.441).

**Table 1:** Biological, morphological, and elastography characteristics of controls and rats with NAFLD.

**Characteristic**	**Control**	**NAFLD**	***P* value**
*n*	15	51	
Platelet count, 10^9^/L	787.5 ± 32.18	828.9 ± 23.41	0.419
Red cell distribution width (%)	14.7 ± 0.12	15.8 ± 0.16	0.041
ALT, IU/L	60.7 ± 2.58	145.5 ± 15.73	0.009
AST, IU/L	208.5 ± 12.34	245.8 ± 18.82	0.333
GGT, IU/L	0.90 ± 0.18	1.91 ± 0.48	0.441
Spleen diameter, cm	3.58 ± 0.01	3.81 ± 0.03	<0.001
PVP measurement, mmHg	4.80 ± 0.03	12.03 ± 0.28	<0.001
LSM, kPa	7.1 (6.6–7.4)	9.1 (7.9–11.0)	<0.001
SSM, kPa	12.8 (12.3–13.3)	15.7 (14.1–17.9)	<0.001

*Data are mean ± standard deviation or median (interquartile range), or number of rats, when appropriate*.

*ALT, alanine aminotransferase; AST, aspartate aminotransferase; GGT, gamma-glutamyl transferase; LSM, liver stiffness measurement; NAFLD, nonalcoholic fatty liver disease; PVP, portal venous pressure; SSM, spleen stiffness measurement*.

### Technical Success and Reliability of 2D-SWE for LSM and SSM

A total of 64 rats with NAFLD underwent liver and spleen 2D-SWE measurements. LSMs were successfully performed on the 64 rats (100%), and all were considered reliable (100%). However, it was successful for SSM in 51 rats (79.7%); SSM obtained by 2D-SWE failed in 8 rats (12.5%) and 5 rats were considered unreliable (7.8%) (Table 2). The success rate of LSM by 2D-SWE was higher than that of SSM (*P* < 0.001).

**Table 2:** Technical success and reliability of LSM and SSM by 2D-SWE in rats with NAFLD.

**Parameter**	**Successful**	**Unsuccessful**	
		**Failure**	**Nonreliable**
LSM	64 (100%)_*_	0	0
SSM	51 (79.7%)	8 (12.5%)	5 (7.8%)

*Data are expressed as number of rats, with percentages in parentheses*.

* *P < 0001; the success rate of LSM by 2D-SWE was higher than that of SSM*.

*LSM, liver stiffness measurement; NAFLD, nonalcoholic fatty liver disease; SSM, spleen stiffness measurement; 2D-SWE, two-dimensional shear wave elastography*.

There was no difference among the median LSM values in rats with NAFLD if they were calculated using three or five measurements: 9.52 (95% confidence interval [CI]: 8.77–10.27) kPa vs. 9.58 (95% CI: 9–10.17) kPa (*P* = 0.904). Similarly, no significant difference was detected among the median SSM values: 15.98 (95% CI: 14.77–17.18) kPa vs. 16.29 (95% CI: 15.32–17.27) kPa (*P* = 0.68). Between the two values calculated using three or five measurements, the concordance was perfect with an ICC of 0.941 (95% CI:0.904–0.964, *P* < 0.001) for LSM and 0.92 (95% CI:0.843–0.958, *P* < 0.001) for SSM.

The intra-operator reliability of LSM and SSM by 2D-SWE was assessed in the 64 and 51 rats with NAFLD and showed technical success. The intra-operator reliability of the five measurements for LSM was excellent, with an ICC of 0.923 (95% CI:0.889–0.949, *P* < 0.001) and a CV of 9.5% (95% CI: 6.7–12.2). The ICC and the CV of the five measurements for SSM were 0.913 (95% CI:0.854–0.95, *P* < 0.001) and 14% (95% CI: 5.2–22.9), respectively, which suggested that the stability of LSM was better than that of SSM.

Based on the above results, the median LSM and SSM values of five 2D-SWE measurements were calculated for further analysis.

### Correlation of Noninvasive Methods With Portal Venous Pressure

**Figure 2:**
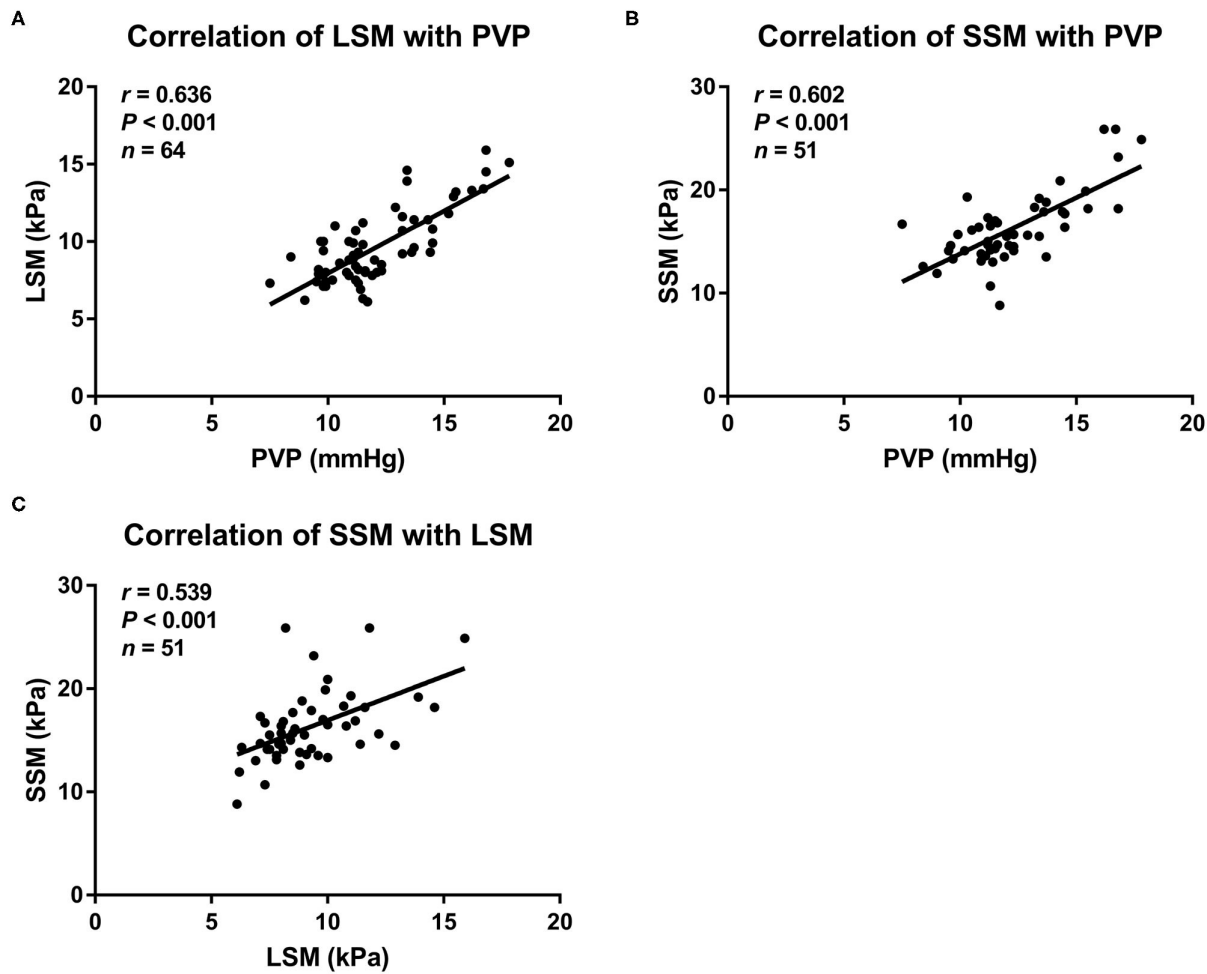
Scatterplots showing correlations between **(A)** LSM, **(B)** SSM, and portal venous pressure, as well as **(C)** LSM with SSM in rat models with NAFLD. LSM, liver stiffness measurement; NAFLD, nonalcoholic fatty liver disease; PVP, portal venous pressure; SSM, spleen stiffness measurement.

LSM and SSM values increase with increase in portal venous pressure of the rats with NAFLD (Figure 2). Among all the noninvasive methods, LSM had the strongest correlation with portal venous pressure values (*r* = 0.636, *P* < 0.001), followed by SSM (*r* = 0.602, *P* < 0.001). At the same time, LSM displayed a positive correlation with SSM in the rats with NAFLD (*r* = 0.539, *P* < 0.001). However, the correlation between the four composite scores (LSPS, PAR, AAR, and APRI) and portal venous pressure was limited.

**Figure 3:**
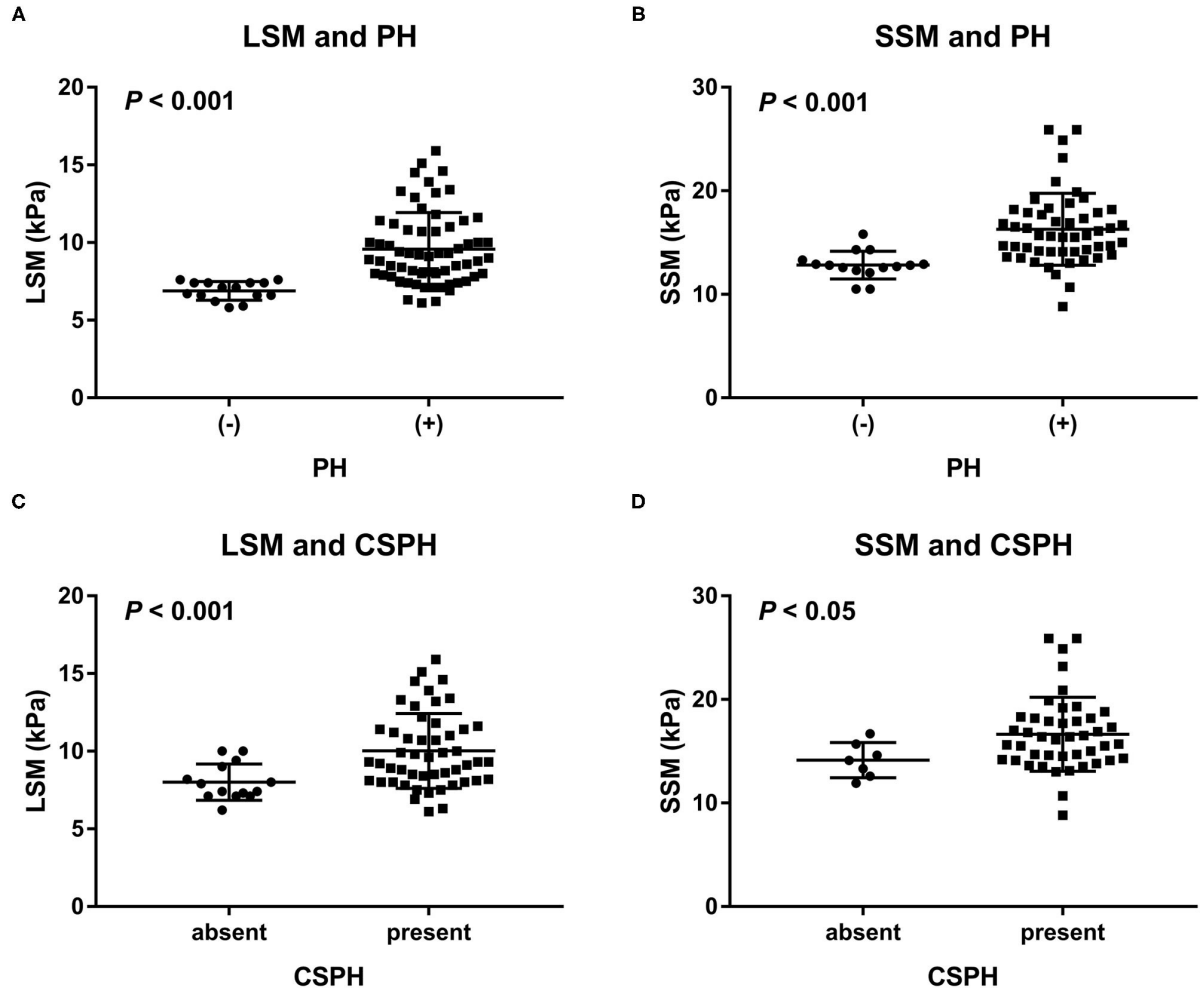
Distribution of **(A)** LSM and **(B)** SSM in rats with and those without PH, as well as distribution of **(C)** LSM and **(D)** SSM in NAFLD rats with and those without CSPH (a portal venous pressure of 10 mmHg or higher). LSM and SSM were significantly higher in the rats with PH than in the rats without PH. Similarly, LSM and SSM were significantly higher in the rats with NAFLD and CSPH than in those with NAFLD but without CSPH. CSPH, clinically significant portal hypertension; LSM, liver stiffness measurement; PH, portal hypertension; SSM, spleen stiffness measurement.

The LSM values were significantly higher in rats with PH than in those without: 9.6 (95% CI: 9–10.2) kPa vs. 6.9 (95% CI: 6.6–7.2) kPa, respectively, (*P* < 0.001). Similarly, the SSM values were also significantly higher in rats with PH than in those without: 16.3 (95% CI: 15.3–7.3) kPa vs. 12.8 (95% CI: 12.1–13.6) kPa, respectively, (*P* < 0.001). The results are shown in Figure 3. Furthermore, the LSM values were significantly higher in the rats with NAFLD, with a portal venous pressure of 10 mmHg or higher, that in those without (10 vs.8 kPa, *P* < 0.001). The same trend was observed for SSM (16.6 vs.14.1 kPa, *P* < 0.05).

### Diagnostic Performance of LSM, SSM, and Composite Scores in Predicting PH

**Figure 4:**
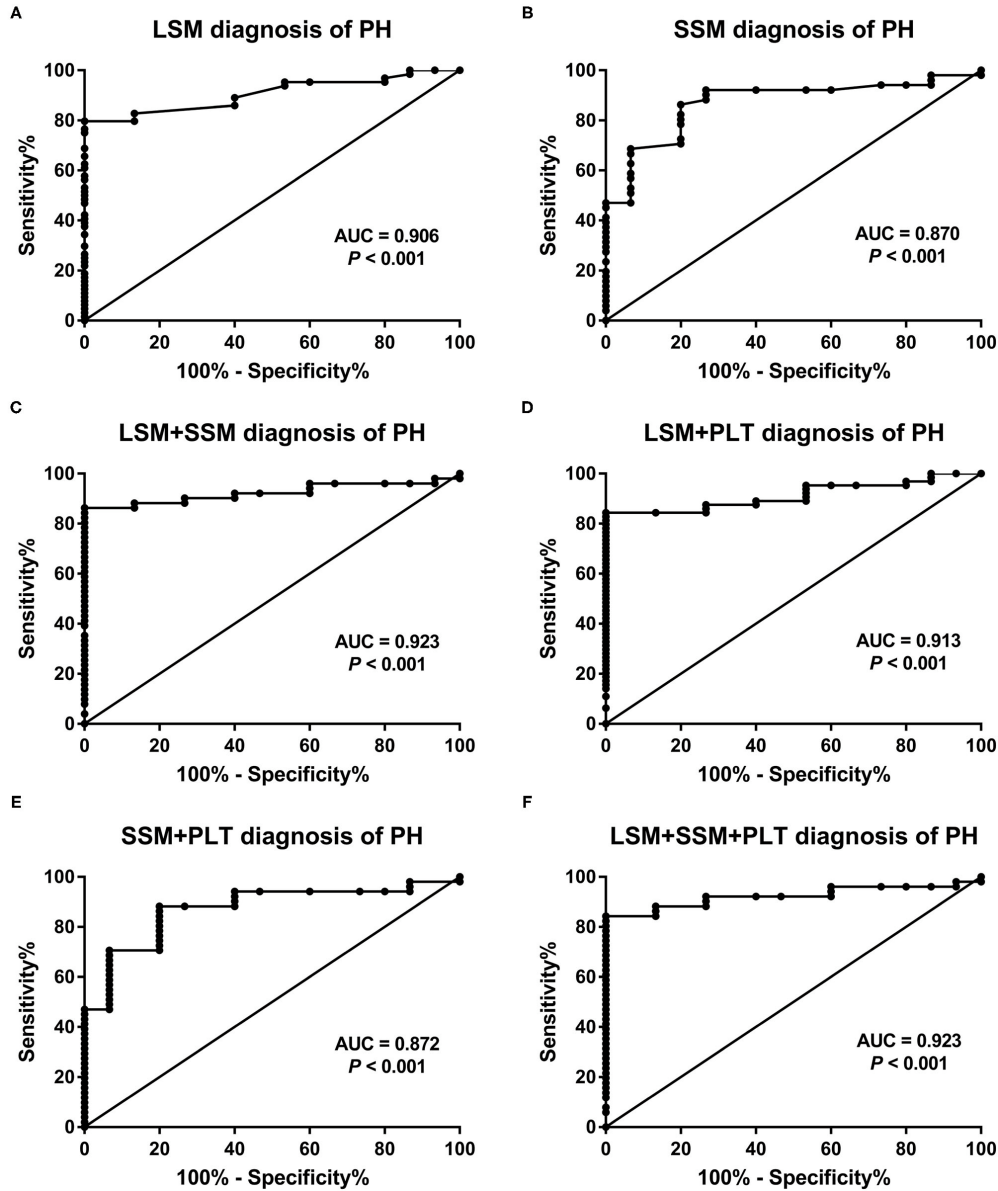
Receiver operating characteristic curves of **(A)** LSM, **(B)** SSM, and **(C–F)** the four combined models for predicting portal venous pressure. AUC, area under the receiver operating characteristic curve; LSM, liver stiffness measurement; PH, portal hypertension; PLT, platelet count; SSM, spleen stiffness measurement.

The AUCs, cutoff values, sensitivity, specificity, PPV, and NPV for the prediction of PH using LSM, SSM, and composite scores are presented in Table 3. In addition, we also investigated four combined models by multivariate logistic analysis. The AUCs of LSM and SSM were 0.906 (95% CI:0.841–0.97) and 0.87 (95% CI:0.776–0.964), respectively, for the diagnosis of PH. Using the Youden index, the cutoff LSM for predicting PH was 7.7 kPa (sensitivity 79.7%, specificity 100%), and the cutoff SSM was 13.4 kPa (sensitivity 86.3%, specificity 80%) (Figure 4). Furthermore, in descending order, the AUCs of LSPS, AAR, APRI, and PSR for predicting PH were 0.793 (95% CI:0.688–0.898),0.668 (95% CI:0.550–0.772),0.533 (95% CI:0.414–0.649), and 0.52 (95% CI:0.366–0.673), respectively. The AUCs of combined models 1 to 4 for the diagnosis of PH were 0.923 (95% CI:0.858–0.988),0.913 (95% CI:0.851–0.974),0.872 (95% CI:0.779–0.965), and 0.923 (95% CI:0.858–0.988), respectively.

**Table 3:** Predictive value of noninvasive methods and combined models for assessing portal venous pressure.

**Noninvasive parameter**	**Portal hypertension**					
	**Cutoff**	**AUC (95% CI)**	**Sensitivity (95% CI, %)**	**Specificity (95% CI, %)**	**PPV (%)**	**NPV (%)**
LSM, kPa	7.7	0.906 (0.841–0.970)	79.7 (67.8–88.7)	100 (78.2–100)	100	53.6
SSM, kPa	13.4	0.870 (0.776–0.964)	86.3 (73.7–94.3)	80.0 (51.9–95.7)	93.6	63.2
LSPS	0.04	0.793 (0.688–0.898)	73.4 (60.9–83.7)	73.3 (44.9–92.2)	92.2	39.3
PSR	197.8	0.520 (0.366–0.673)	37.5 (25.7–50.5)	86.7 (59.5–98.3)	92.3	24.5
AAR	3.18	0.668 (0.550–0.772)	76.7 (64.0–86.6)	60.0 (32.3–83.7)	88.5	39.1
APRI	0.23	0.533 (0.414–0.649)	50.0 (36.8–63.2)	73.3 (44.9–92.2)	88.2	26.8
Combined model 1^†^	0.73	0.923 (0.858–0.988)	86.3 (73.7–94.3)	100 (78.2–100)	100	58.2
Combined model 2^‡^	0.74	0.913 (0.851–0.974)	84.4 (73.1–92.2)	100 (78.2–100)	100	60.0
Combined model 3^§^	0.65	0.872 (0.779–0.965)	88.2 (76.1–95.6)	80.0 (51.9–95.7)	93.8	66.6
Combined model 4^‡^	0.71	0.923 (0.858–0.988)	84.3 (71.4–92.3)	100 (78.2–100)	100	65.2

† *combined model 1 represents LSM and SSM combined model*;

‡ *combined model 2 represents LSM and platelet count combined model*;

§ *combined model 3 represents SSM and platelet count combined model; ‡, combined model 4 represents LSM and SSM, platelet count combined model*.

*AAR, aspartate aminotransferase/alanine aminotransferase ratio; APRI, aspartate aminotransferase-to-platelet count ratio index; AUC, area under the receiver operating characteristic curve; CI, confidence interval; LSM, liver stiffness measurement; LSPS, liver stiffness-spleen diameter to platelet ratio score; NPV, negative predictive value; PPV, positive predictive value; PSR, platelet count/spleen diameter ratio; SSM, spleen stiffness measurement*.

When comparing the AUCs, the performance of LSM in the diagnosis of PH was significantly higher than that of LSPS (*P* = 0.047), AAR (*P* < 0.001), APRI (*P* < 0.001), and PSR (*P* < 0.001). However, there was no significant difference between LSM and SSM in evaluating PH (*P* = 0.618). Among the four composite scores, LSPS had higher performance than APRI and PSR in assessing PH (all *P* < 0.001), and no significant difference between LSPS and AAR was found (*P* = 0.167). The AUCs of combined models 1 to 4 for the assessment of PH were more than 085, with no significant differences among the combined models (all *P* > 0.05). The AUCs of combined models 1, 2, and 4 in the assessment of PH were >0.9, and no significant differences were found among the three combined models (all *P* > 0.05). Our results also showed that the AUCs of combined models 1, 2, and 4 were slightly higher than those of LSM, but that the differences were not statistically significant (all *P* > 0.05). Furthermore, the four combined models had higher AUC values than SSM (AUC = 0.872–0.923 vs. AUC = 0.87, all *P* > 0.05).

## Discussion

In this study, we revealed the value of LSM and SSM obtained by 2D-SWE, the four composite scores (LSPS, PSR, AAR, and APRI), and the four combined models, namely, combined model 1 (LSM and SSM), combined model 2 (LSM and PLT), combined model 3 (SSM and PLT), and combined model 4 (LSM, SSM and PLT), for predicting PH in rat models with NAFLD. Our study demonstrated that both LSM and SSM obtained by 2D-SWE showed a positive correlation with portal venous pressure and exhibited higher diagnostic accuracy for assessing PH in NAFLD compared with the four composite scores. In addition, the diagnostic performance of combined models 1, 2, and 4 were similar and slightly higher than that of LSM, but the differences were not significant.

There is an urgent need to develop alternative, noninvasive methods for detecting PH, and as such, it will be very important for early diagnosis and predictive significance (27). Some studies have explored the value of LSM obtained by 2D-SWE in diagnosing PH in recent years. However, only few previous studies have involved patients with NAFLD. A prospective study by Jeon et al. (28) demonstrated that the AUC of LSM was 0.818 for the diagnosis of clinically significant PH in patients with hepatitis B-related liver disease. Another study reported that the AUCs of LSM were 0.72 and 0.77 for diagnosing clinically significant PH and severe PH, respectively, in patients with hepatitis B-related cirrhosis (8). In our study, LSM was significantly increased in the rat models with NAFLD when compared to those in controls and positively correlated with portal venous pressure, with a correlation coefficient of 0.636 (*P* < 0.001), which was relatively higher than that of a previous study (*r* = 0.607) (8). Moreover, LSM showed a good diagnostic value for evaluating PH, with an AUC of more than 0.9. Our results were higher than those of previous studies (8, 28). This discrepancy may be due to the development of PH influenced by a pattern of fibrosis in the liver specific to the etiology of chronic liver disease. According to the etiology, PH is likely to have a different onset (12), for instance, in patients with nonalcoholic steatohepatitis, which may develop PH even in pre-cirrhotic stages (29).

The hemodynamics and morphologic characteristics of the spleen are subsequently changed when chronic liver disease progresses (30). PH can cause splenic congestion, which increases the stiffness of splenic tissue (31). Our study showed that SSM had a moderately strong correlation with portal venous pressure values (*r* = 0.602, *P* < 0.001), and exhibited a good performance that was comparable to that of LSM in terms of detecting PH (AUC = 0.87 vs. AUC = 0.906, *P* > 0.05). Furthermore, compared to LSM, SSM displayed a relatively higher sensitivity for evaluation of PH. Altogether, both LSM and SSM obtained by 2D-SWE can be used as promising noninvasive parameters for the diagnosis of PH in NAFLD. However, we found that the success and stability rates of SSM were relatively lower than those of LSM. In our study, we could not obtain satisfactory SSM results (including failed and unreliable) in 13 rats with NAFLD, accounting for 20.3% (13/64) of all the rats with NAFLD. This was mainly due to two factors: small spleen size and colonic gas, which affect the visualization of the spleen and the clarity of its image, and result in poor sonic window of the spleen. The stability of SSM may also be influenced by cardiac beat-induced modifiable movements. Similarly, a recent study by Jeon et al. (28) reported that the failure and unreliable results of SSM were more frequent than those of LSM. These results, therefore, indicated that LSM seemed to be more reliable and useful for the evaluation of PH in NAFLD than SSM given the technical success and stability results.

Previously, Elkrief et al. (32) have reported that SSM did not achieve satisfactory results in the diagnosis of clinically significant PH (AUC = 0.64). Of note, the population in this study was composed of patients with predominantly decompensated cirrhosis (Child-Pugh C 44%) and severe PH (median HVPG = 17 mmHg). Sharma et al. (22) found that SSM did not show a correlation with HVPG in twenty-four patients with more severe PH (HVPG ≥ 19 mmHg). In these previous studies, the unsatisfactory results of SSM may be due to the effect of various shunts arising during PH progression (33). Further studies focusing on the diagnostic superiority of SSM over LSM obtained by 2D-SWE for evaluation of PH in patients with compensated chronic liver disease are warranted.

LSPS, PSR, AAR, and APRI are common composite scores. Zhu et al. (8) showed that the AUC of LSPS was 0.76 for assessing clinically significant PH and 0.8 for assessing severe PH. In Elkrief's study (32), the AUC of LSPS (by 2D-SWE) was 0.76 for diagnosis of clinically significant PH. In our study, we found that the correlation between the four composite scores and portal venous pressure was limited. Among these, LSPS had better diagnostic performance, with an AUC of 0.793, which was slightly higher than that reported in previous studies. LSPS combines LSM, spleen diameter, and PLT; however, its value was not better than that of LSM alone for diagnosing PH. In contrast, the performance of LSM was superior to that of LSPS in our study. Besides, PSR and APRI could not display satisfactory results in the diagnosis of PH. Initially, PSR was proposed as a noninvasive parameter for predicting esophageal varices (21). Nevertheless, a previous study has confirmed that PSR was unable to distinguish between patients with large esophageal varices and those with small ones, and that its accuracy in diagnosing the presence of esophageal varices was also lower than that of LSM (obtained by TE) (22). Blood parameters, such as PLT, AST, and ALT, may be influenced by extrahepatic lesions (30); AAR, combining AST and ALT, was not reliable enough to accurately evaluate PH. Therefore, 2D-SWE measurements including LSM and SSM may be more advantageous for diagnosing PH in NAFLD than composite scores.

Furthermore, to improve the accuracy of 2D-SWE measurements, we attempted to study four combined diagnostic models and compared LSM and SSM with the combined diagnostic models. In our study, combined models 1, 2, and 4 showed a similar diagnostic value that was slightly higher than that of combined model 3, although the difference was not significant. In addition, between the four combined models and the single-measurement methods (LSM and SSM), no significant differences were found. The combined models may be too complex in clinical practice. Hence, it appears that LSM is both a convenient and dependable noninvasive diagnostic tool for evaluation of PH.

Some limitations are worth considering in this study. First, the unsuccessful result of SSM was explicitly higher than that of LSM in our rat models as well as in clinical samples reported in previous studies (8, 28). It is principally because of small spleen size and colonic gas. To improve the technical success results of SSM, further studies are required. It is worth mentioning that 2D-SWE as a novel elastography technique has a significantly higher rate of success and reliability for the measurement of SSM than TE (32). Second, portal venous pressure was measured under aseptic conditions in an operation room by experienced researchers after the rats were anesthetized. There may be a difference in the portal venous pressure measurement between animals in the conscious state and those in the anesthetized state. Third, the optimal cutoff values of LSM and SSM obtained by 2D-SWE for evaluating PH in subjects with NAFLD should be determined. Of course, it needs further studies. Finally, our study used rat models of NAFLD. Additional studies with a population of patients with NAFLD are required to confirm our results. Moreover, only male animals were used in our study. Future efforts are required to address gender disparities.

In conclusion, LSM and SSM obtained by 2D-SWE had notably better diagnostic performance in evaluating PH in the rat models with NAFLD than the composite scores such as LSPS, PSR, AAR, and APRI. Considering the technical success and stability results, LSM seemed to be more reliable and useful than SSM. The four combined models, compared to LSM and SSM, did not significantly improve diagnostic accuracy in evaluating PH.

## Data Availability Statement

The raw data supporting the conclusions of this article will be made available by the authors, without undue reservation.

## Ethics Statement

The animal study was reviewed and approved by Institutional Animal Care and Use Committee (IACUC) of The Second Affiliated Hospital of Fujian Medical University.

## Author Contributions

BD collected the data and wrote the first draft of the manuscript. YPC provided the pathological scores of liver tissues. BD, YJC, and RQ performed the statistical analysis. GL conceived of and supervised the study. All the authors edited the manuscript, provided intellectual input, contributed to the article and approved the submitted version.

## Funding

This study was supported by the Startup Fund for Scientific Research, Fujian Medical University (2020QH2037).

## Conflict of Interest

The authors declare that the research was conducted in the absence of any commercial or financial relationships that could be construed as a potential conflict of interest.

## Publisher's Note

All claims expressed in this article are solely those of the authors and do not necessarily represent those of their affiliated organizations, or those of the publisher, the editors and the reviewers. Any product that may be evaluated in this article, or claim that may be made by its manufacturer, is not guaranteed or endorsed by the publisher.

## References

[1] Cotter TG Rinella M. Nonalcoholic fatty liver disease 2020: the state of the disease. Gastroenterology. (2020) 158:1851–64. 10.1053/j.gastro.2020.01.052 32061595

[2] Li J Zou B Yeo YH Feng Y Xie X Lee DH. Prevalence, incidence, and outcome of non-alcoholic fatty liver disease in Asia, 1999-2019: a systematic review and meta-analysis. Lancet Gastroenterol Hepatol. (2019) 4:389–98. 10.1016/S2468-1253(19)30039-1 30902670

[3] Monserrat-Mesquida M Quetglas-Llabrés M Abbate M Montemayor S Mascaró CM Casares M. Oxidative stress and pro-inflammatory status in patients with non-alcoholic fatty liver disease. Antioxidants (Basel). (2020) 9:759. 10.3390/antiox9080759 32824349 PMC7463614

[4] Li J Sehrawat TS Chen J Hilscher MB Glaser KJ Arab JP. Quantitative assessment of portal hypertension with bi-parametric dual-frequency hepatic MR elastography in mouse models. Eur Radiol. (2021) 31:2303–11. 10.1007/s00330-020-07341-3 33026502 PMC7981248

[5] Feng Q Guan S Zhao JR Zhao XY Zhang CC Wang L. Gadobenate dimeglumine-enhanced magnetic resonance imaging can accurately predict the severity of esophageal varices and portal vein pressure in patients with hepatitis B cirrhosis. J Dig Dis. (2020) 21:104–11. 10.1111/1751-2980.12843 31922658

[6] Singh R Wilson MP Katlariwala P Murad MH McInnes MDF Low G. Accuracy of liver and spleen stiffness on magnetic resonance elastography for detecting portal hypertension: a systematic review and meta-analysis. Eur J Gastroenterol Hepatol. (2021) 32:237–45. 10.1097/MEG.0000000000001724 32282542

[7] de Franchis R Baveno VI. Faculty Expanding consensus in portal hypertension: report of the Baveno VI Consensus Workshop: stratifying risk and individualizing care for portal hypertension. J Hepatol. (2015) 63:743–52. 10.1016/j.jhep.2015.05.022 26047908

[8] Zhu YL Ding H Fu TT Peng SY Chen SY Luo JJ. Portal hypertension in hepatitis B-related cirrhosis: Diagnostic accuracy of liver and spleen stiffness by 2-D shear-wave elastography. Hepatol Res. (2019) 49:540–9. 10.1111/hepr.13306 30597744

[9] Pons M Augustin S Scheiner B Guillaume M Rosselli M Rodrigues SG. Noninvasive diagnosis of portal hypertension in patients with compensated advanced chronic liver disease. Am J Gastroenterol. (2021) 116:723–32. 10.14309/ajg.0000000000000994 33982942

[10] Wan S Liu X Jiang H Teng Z Song B. Noninvasive imaging assessment of portal hypertension: where are we now and where does the future lie? Expert Rev Mol Diagn. (2021) 21:343–5. 10.1080/14737159.2021.1904897 33749473

[11] Agbim U Asrani SK. Non-invasive assessment of liver fibrosis and prognosis: an update on serum and elastography markers. Expert Rev Gastroenterol Hepatol. (2019) 13:361–74. 10.1080/17474124.2019.1579641 30791772

[12] Roccarina D Rosselli M Genesca J Tsochatzis EA. Elastography methods for the non-invasive assessment of portal hypertension. Expert Rev Gastroenterol Hepatol. (2018) 12:155–64. 10.1080/17474124.2017.1374852 28856972

[13] Barr RG. Shear wave liver elastography. Abdom Radiol (NY). (2018) 43:800–7. 10.1007/s00261-017-1375-1 29116341

[14] Wei H Jiang HY Li M Zhang T Song B. Two-dimensional shear wave elastography for significant liver fibrosis in patients with chronic hepatitis B: a systematic review and meta-analysis. Eur J Radiol. (2020) 124:108839. 10.1016/j.ejrad.2020.108839 31981878

[15] Thiele M Hugger MB Kim Y Rautou PE Elkrief L Jansen C. 2D shear wave liver elastography by Aixplorer to detect portal hypertension in cirrhosis: an individual patient data meta-analysis. Liver Int. (2020) 40:1435–46. 10.1111/liv.14439 32180327

[16] Zhuang Y Ding H Zhang Y Sun H Xu C Wang W. Two-dimensional shear-wave elastography performance in the noninvasive evaluation of liver fibrosis in patients with chronic hepatitis B: comparison with serum fibrosis indexes. Radiology. (2017) 283:873–82. 10.1148/radiol.2016160131 27982760

[17] Chen S Jiang T. Preoperative noninvasive assessment for liver fibrosis in hepatocellular carcinoma patients with chronic hepatitis B: comparison of two-dimensional shear-wave elastography with serum liver fibrosis models. Eur J Radiol. (2020) 133:109386. 10.1016/j.ejrad.2020.109386 33160197

[18] Liu J Li Y Yang X Ji Y Zhang Y Wan Q. Comparison of two-dimensional shear wave elastography with nine serum fibrosis indices to assess liver fibrosis in patients with chronic hepatitis B: a prospective cohort study. Ultraschall Med. (2019) 40:237–46. 10.1055/a-0796-6584 30630194

[19] Dong B Lyu G Chen Y Lin G Wang H Qin R. Comparison of two-dimensional shear wave elastography, magnetic resonance elastography, and three serum markers for diagnosing fibrosis in patients with chronic hepatitis B: a meta-analysis. Expert Rev Gastroenterol Hepatol. (2021) 15:1077–89. 10.1080/17474124.2021.1880894 33487039

[20] Kim BK Han KH Park JY Ahn SH Kim JK Paik YH. A liver stiffness measurement-based, noninvasive prediction model for high-risk esophageal varices in B-viral liver cirrhosis. Am J Gastroenterol. (2010) 105:1382–90. 10.1038/ajg.2009.750 20087336

[21] Giannini E Botta F Borro P Risso D Romagnoli P Fasoli A. Platelet count/spleen diameter ratio: proposal and validation of a non-invasive parameter to predict the presence of oesophageal varices in patients with liver cirrhosis. Gut. (2003) 52:1200–5. 10.1136/gut.52.8.1200 12865282 PMC1773759

[22] Sharma P Kirnake V Tyagi P Bansal N Singla V Kumar A. Spleen stiffness in patients with cirrhosis in predicting esophageal varices. Am J Gastroenterol. (2013) 108:1101–7. 10.1038/ajg.2013.119 23629600

[23] Colecchia A Montrone L Scaioli E Bacchi-Reggiani ML Colli A Casazza G. Measurement of spleen stiffness to evaluate portal hypertension and the presence of esophageal varices in patients with HCV-related cirrhosis. Gastroenterology. (2012) 143:646–54. 10.1053/j.gastro.2012.05.035 22643348

[24] Königshofer P Brusilovskaya K Schwabl P Reiberger T. Animal models of portal hypertension. Biochim Biophys Acta Mol Basis Dis. (2019) 1865:1019–30. 10.1016/j.bbadis.2018.07.018 30055295

[25] Dietrich CF Bamber J Berzigotti A Bota S Cantisani V Castera L. EFSUMB guidelines and recommendations on the clinical use of liver ultrasound elastography, Update 2017 (Long Version). Ultraschall Med. (2017) 38:e16–47. 10.1055/s-0043-103952 28407655

[26] Bedossa P Poitou C Veyrie N Bouillot JL Basdevant A Paradis V. Histopathological algorithm and scoring system for evaluation of liver lesions in morbidly obese patients. Hepatology. (2012) 56:1751–9. 10.1002/hep.25889 22707395

[27] Ding XC Ma WL Li MK Liu SW Liu XY Hai L. A meta-analysis of the value of vWF in the diagnosis of liver cirrhosis with portal hypertension. J Clin Transl Hepatol. (2019) 7:3–8. 10.14218/JCTH.2018.00036 30944812 PMC6441641

[28] Jeon SK Lee JM Joo I Yoon JH Lee DH Han JK. Two-dimensional shear wave elastography with propagation maps for the assessment of liver fibrosis and clinically significant portal hypertension in patients with chronic liver disease: a prospective study. Acad Radiol. (2020) 27:798–806. 10.1016/j.acra.2019.08.006 31494001

[29] Francque S Verrijken A Mertens I Hubens G Van Marck E Pelckmans P. Noncirrhotic human nonalcoholic fatty liver disease induces portal hypertension in relation to the histological degree of steatosis. Eur J Gastroenterol Hepatol. (2010) 22:1449–57. 10.1097/MEG.0b013e32833f14a1 21389796

[30] Wang XP Wang Y Ma H Wang H Yang DW Zhao XY. Assessment of liver fibrosis with liver and spleen magnetic resonance elastography, serum markers in chronic liver disease. Quant Imaging Med Surg. (2020) 10:1208–22. 10.21037/qims-19-849 32550131 PMC7276364

[31] Barr RG Wilson SR Rubens D Garcia-Tsao G Ferraioli G. Update to the society of radiologists in ultrasound liver elastography consensus statement. Radiology. (2020) 296:263–74. 10.1148/radiol.2020192437 32515681

[32] Elkrief L Rautou PE Ronot M Lambert S Dioguardi Burgio M Francoz C. Prospective comparison of spleen and liver stiffness by using shear-wave and transient elastography for detection of portal hypertension in cirrhosis. Radiology. (2015) 275:589–98. 10.1148/radiol.14141210 25469784

[33] Zykus R Jonaitis L Petrenkiene V Pranculis A Kupcinskas L. Liver and spleen transient elastography predicts portal hypertension in patients with chronic liver disease: a prospective cohort study. BMC Gastroenterol. (2015) 15:183. 10.1186/s12876-015-0414-z 26702818 PMC4690243

